# Normative and limit values of speed, endurance and power tests results of young football players

**DOI:** 10.3389/fphys.2024.1502694

**Published:** 2025-01-08

**Authors:** Michał Nowak, Marta Szymanek-Pilarczyk, Artur Stolarczyk, Łukasz Oleksy, Jarosław Muracki, Jacek Wąsik

**Affiliations:** ^1^ Faculty of Physical Culture Sciences, Collegium Medicum im. dr. Władysława Biegańskiego, Jan Długosz University in Częstochowa, Częstochowa, Poland; ^2^ Science Department of the RKS Raków Częstochowa Academy, Częstochowa, Poland; ^3^ Faculty of Physical Culture Sciences, Jan Długosz University in Częstochowa, Częstochowa, Poland; ^4^ Department of Orthopaedics and Rehabilitation, Medical and Dentistry Faculty, Medical University of Warsaw, Warsaw, Poland; ^5^ Department of Physiotherapy, Faculty of Health Sciences, Jagiellonian University Medical College, Kraków, Poland; ^6^ Department of Orthopedics, Traumatology and Hand Surgery, Faculty of Medicine, Wrocław Medical University, Wrocław, Poland; ^7^ Department of Physical Culture and Health, Institute of Physical Culture Sciences, University of Szczecin, Szczecin, Poland

**Keywords:** endurance, speed, maximal aerobic speed, young soccer players, physical demands

## Abstract

**Introduction:**

This study aimed to assess the development of speed, endurance and power in young football players and to create percentile charts and tables for standardized assessment.

**Methods:**

Cross-sectional data were collected from 495 male players aged 12–16 years at RKS Raków Częstochowa Academy in 2018–2022. Players participated in a systematic training in which running time 5 m, 10 m, 30 m, lower limb power (standing long jump), and Maximum Aerobic Speed (MAS) were measured using the 30–15 Intermittent Fitness Test. All tests were performed under constant environmental conditions by qualified personnel. Statistical analysis included ANOVA and percentile distribution for P3, P10, P25, P50, P75, P90, P97.

**Results:**

Results indicated that the most significant improvements occurred between the ages of 13 and 14, with increased speed over all distances and a significant increase in power. Percentile tables were developed, highlighting improvements in speed 5 m: 0.087–0.126 s; 10 m 0.162–0.215 s; 30 m: 0.438–0.719 s and power in the long jump test: 31–48 cm. Improvements in MAS ranged from 0.3 to 0.6 m/s across the percentiles.

**Discussion:**

The results highlight the need for individual training programs tailored to the biological maturity of players. The developed percentile charts and tables offer a valuable tool for coaches and sports scientists to monitor progress, optimize training loads, and minimize the risk of injury, providing a frame of reference for assessing the physical development of young soccer players. Future research should focus on extending these charts and tables to other age groups and genders to refine training methodologies further.

## Introduction

Football is one of the most popular sports played by children and teenagers worldwide. The development of this discipline affects many areas - social, scientific, educational, and economic, giving them an impulse for development. The involvement of young adepts and the popularity of football remains at a very high level (Toukabri and Toukabri, 2023). This, in turn, affects the work related to raising the level of skills of all people participating in systematic training processes (Chambers, 1991). The most important goal of the game of football is to score more goals than the opponent (Shan, 2023). An element that significantly affects this fact, apart from tactical and technical training, is motor preparation. It consists of, among other things, power, aerobic and anaerobic capacity - all at least at a high level (Clemente et al., 2020). Currently, the changing dynamics of the game force the preparation of training measures based on sub-maximum or maximum values, most often in total per week exceeding the total load values in relation to the actual conditions set by the opponent during the match competition.

Speed, motor coordination, or correct decision-making, as well as the intention (purposefulness) of using a given training measure (exercise), affect the development of a young player in the long run (Erikstad et al., 2018). Speed in football can be divided into several categories, e.g., the speed of movement of a player with the ball, without the ball, and the speed of decision-making. Each of these categories can be equally important in training processes. It is suggested that actions on the pitch based on speed directly affect the success or failure of the team (Milenkovic, 2011). In the literature, researchers most often analyze running speed over repetitive distances in the range of 5–55 m (Nicholson et al., 2021). In Poland’s categories of children and adolescents, tests on very short distances, i.e., 5 m, 10 m, and 30 m, are accepted as the gold standard (Andrzejewski et al., 2009; 2013; Gardašević et al., 2016). Researchers indicate that the development process of a young athlete should be planned comprehensively, taking into account various motor skills (Duggan et al., 2022). Current requirements in soccer include multiple high-intensity efforts. One of the key objectives of the training process is to prepare players to maintain a state of readiness that allows them to effectively create activities characterized by high or very high intensity during the entire match (Duthie et al., 2018).

Scientists and coaches confirm the thesis that a high level of endurance is fundamental for achieving outstanding sports results (Clemente et al., 2021; 2024). This allows players to implement various advanced tactical concepts (Hoppe et al., 2013; Perroni et al., 2023). The speeds achieved by athletes in progressive tests with breaks, such as the Beep Test, Yo-Yo, or the 30–15 test, are correlated with the speeds at which the athletes achieve maximum oxygen consumption (VO2max) (Magee et al., 2021; Quan et al., 2024). They also correlate with the level of intensity observed during real matches (Krustrup et al., 2003). This confirms the important role of specific endurance training, which not only affects performance but also the ability to regenerate quickly. Therefore, it is an integral part of modern football training programs (Clemente et al., 2024).

Another important feature in a footballer’s training is to increase power by shaping, e.g., the explosive strength of the lower limbs (Szymanek-Pilarczyk et al., 2023; Wang et al., 2023). This can be observed in activities such as acceleration, changes of direction, and performing repeated high-intensity efforts (De Salles et al., 2012). The results of the study indicate a negative correlation between the peak power and height of the Countermovement Jump (CMJ) and the sprint time obtained at different distances (Bartosz et al., 2024; Gisladottir et al., 2024; Shan, 2023). In coaching practice, the importance of the development of explosive power of the lower limbs is emphasized in terms of direct impact on the effectiveness and efficiency of actions on the pitch and, consequently, on achieving the final success in football (Wang et al., 2023).

Training programs for children and adolescents should take into account the gradual and harmonious development of all motor skills. A modified wave periodization model was used in the training system at the RKS Raków Academy. A long-term plan for the development of the players’ motor skills was developed. Systematic monitoring of selected motor parameters of players allowed us to create a new tool, growth charts. Growth charts are a statistical tool used to assess and monitor children’s physical development by comparing their height, weight, and other parameters with those of their peers. They help specialists identify possible health problems and monitor the stability of the child’s development. Their usefulness lies in enabling standardized assessment of development in different populations and comparing growth patterns at the global level. Regular use of growth charts makes it possible to detect deviations from the norm early and take appropriate medical interventions. They are a key tool in pediatrics that helps to provide children with optimal healthcare around the world. To the best of authors’ knowledge there are no percentile charts for results of speed, endurance and power tests of youth elite and subelite soccer players described in scientific literature. In the case of our research, this is the first attempt to show that this type of data can also be used in work with young athletes.

The study’s aim was to learn about the changes in the adaptive motor skills of children and adolescents participating in the RKS Raków Academy training program and to build tools for assessing the development of selected motor skills for young footballers. This allowed for the development of standardized reference curves, the so-called percentile charts, on the basis of data collected from 5-year studies for selected physical fitness indicators such as: running speed at 5 m, 10 m, 30 m, power and MAS index among boys training football at the RKS Raków Częstochowa Academy.

The application of this research could be to create a unified benchmark that will enable coaches and scientists to monitor and evaluate athletes’ progress more accurately. Growth charts can be a tool that supports the optimization of the training process, improvement of sports performance, and reduction of the risk of injury, which in the long term will contribute to raising the sports level of young footballers. At the same time, placing individual results on the growth chart will allow you to estimate the potential for developing a given motor trait and give tips for working with the athlete.

## Material and methods

### Subjects

Cross-sectional studies of selected physical fitness indicators were carried out in 2018–2022 among football players of the RKS Raków Częstochowa Academy aged 12–16. The sporting level of the examined players was Tier 3 according to McKay’s classification (McKay et al., 2022). The training experience of the respondents ranged from 4 to 8 years. During each of the analysed seasons the players competed in the highest possible junior league. The inclusion criteria were: i) age from 12 to 16 accordingly to the age groups; ii) being a male football player of the academy; iii) training experience over 4 years. The exclusion criteria were: i) serious injury or illness resulting in time loss over 1 month in the year period of the study; ii) illness or injury before or at the moment of testing. The analyzed group of players amounted to a total of 495 young healthy male football players. Considering age ranges, it was divided into five subgroups. The percentage distribution was: 16-year-olds – 19.8% of the respondents, 15-year-olds – 20.6%, 14-year-olds – 20.4%, 13-year-olds – 19.6% and 12-year-olds–also 19.6%. During the monitored period of 4 years there was a rotation of players, which is natural in the youth football academy, but it did not exceed 5% overall. The anthropometric characteristics of the respondents are presented in Table 1. The subjects participated in systematic sports training developed according to the wave periodization program developed following the tactical periodization work model (Szymanek-Pilarczyk et al., 2023; Szymanek-Pilarczyk et al., 2024a; Szymanek-Pilarczyk et al., 2024b).

**TABLE 1:** The characteristics of the study group are divided into age categories.

Age (years)	Number of players	Body height (cm) ± SD	Body weight (kg) ± SD	Body fat (%) ± SD	Muscle mass (kg) ± SD
16	98	178 ± 3.89	69.02 ± 5.17	15.15 ± 1.97	54.75 ± 4.18
15	102	178 ± 4.71	67.20 ± 5.42	15.22 ± 1.92	54.05 ± 4.42
14	101	172 ± 7.00	59.30 ± 6.78	14.99 ± 2.39	47.74 ± 4.73
13	97	165 ± 9.42	51.27 ± 8.91	15.15 ± 2.69	40.89 ± 6.82
12	97	154 ± 4.59	42.83 ± 7.11	19.24 ± 6.62	32.12 ± 4.71

SD, standard deviation.

The simplified training load scheme in each working week consisted of 6 training units on the pitch (4 team training x 90 min, 1 formation training including profiling of players in positions x 60 min, 1 match x 45–90 min), and 2 training sessions in the gym lasting 2 × 45 min (1 session—upper muscle parts and 2 sessions—lower muscle parts). All age categories in the described period were trained according to a uniform training plan. Every training session started with the warm-up, general development, specific preparation focused on selected motor abilities considering day-to-match and with accordance with the training plan. The methods and forms used in the training process were adjusted to the age and level of the participants (Szymanek-Pilarczyk et al., 2023; Szymanek-Pilarczyk et al., 2024a; Szymanek-Pilarczyk et al., 2024b).

### Ethics

All participants were thoroughly informed about the content of the study, its objectives, possible risks, and benefits. When joining the club and renewing the declarations of participation in the academy every player signed an informed consent to participate in research analysing their monitoring data for scientific purposes and publication. This study’s tasks and tests were typically performed during training (sprints, runs, and jumps). All participants had a federation license, based on which their parents signed a document at the beginning of the season authorizing them to participate in the club’s football activities. This type of intervention does not alter standard soccer training or involve motor activities different from regular training and matches. Therefore, the intervention has never posed an additional risk beyond the threat associated with ordinary football practice. Moreover, all participants underwent a medical examination before the start of the season, and the tests were carried out without injury or physical discomfort. The study was in line with the requirements of the Declaration of Helsinki. Approval was obtained from the bioethics committee at the District Medical Chamber in Krakow (approval number: No. 35/KBL/OIL/2024; approval date: 24 April 2024).

### Procedures

The tests were always carried out in the same period and place–a sports hall with a synthetic surface and a temperature of about 20°C. The competitors performed the tests in sports shoes with an indoor sole. The measurements took place at the same times of the day in dedicated time intervals. 1 h per team. During this period, the measurements were taken by permanently qualified personnel, i.e., the training staff consisting of motor preparation trainers (qualifications confirmed by the international certificate of the Australian Strength and Conditioning Association (ASCA). All players training at the Academy took part in the tests. The exclusion criterion was an injury, illness, or other infection that prevented the test from being performed at 100% of the athlete’s capacity. The tests were always carried out in the same microcycle on the day referred to in terminology as the third day after the match (MD+3). The training before the tests was of a low-intensity regenerative nature. The tests were always performed in June, after the end of the season in the same order: 1) warm-up, 2) speed test (1^st^ attempt), rest 5 min, 3) long jump (1^st^ attempt), rest 5 min, 4) speed test (2^nd^ attempt), rest 5 min, 5) long jump (2^nd^ attempt), rest 10 min, 6) endurance test.

### RAMP warm-up

The warm-up lasted 12 min. The first part of the warm-up lasted 4 min and aimed to raise the body temperature Raise – (R) through running exercises performed at 15 m. In this part, the following were performed: jogging, running combined with swings/arm circles, stepping out–extended sideways and forward with a variable rhythm, abduction and adduction of legs in a free rhythm, skips A, B, C, D performed at a moderate speed. The second part of the warm-up was aimed at muscle activation and increasing the range of motion of both muscles and joints Activate – (A), Mobilise – (M). In this block, one series of 12 repetitions of exercises were used: Squat, Lunge forward, Lunge to the side, Hip Thrust, Single-Leg Romanian Deadlift (RDL SL), Push Up, Abdominal exercises, Back exercises - torso raises, and rowing. Exercises to increase the range of motion were carried out in the form of dynamic stretching of all muscle groups, taking into account many planes. The described part lasted 6 min. The last part of the warm-up was about increasing the speed potential of the Potentiate muscles – (P) (Jeffreys, 2017). Six runs were made on a 15 m section. Before the run, the competitors performed specific exercises (time = 3 s) with maximum speed and commitment. These were jumping jacks, fast feet, pogo jumps, and burpees. This part of the warm-up lasted 2 min.

### Test protocols

#### Speed

The measurement of running speed took place in a straight line. The speed was recorded at distances of 5, 10, and 30 m. For this purpose, electronic measuring equipment was used–FITLIGHT^®^ photocells (app version 3.2.6i, Ontario, Canada). The athlete’s foot was 20 cm in front of the line of the first photocell. The start started from a high, passive position without torso rotation (swing of the arms). The photocells were placed 95 cm above the surface. The runner decided about the moment of the start himself. Each competitor made two attempts, and the best result was recorded with an accuracy of 0.001 s. The resting break between rehearsals was 3 min.

#### Power

The power test involved measuring the length of the long jump from a standstill using a tape measure with an accuracy of 0.01 m. The subjects performed a two-legged jump from the spot with an arms swing. The competitors made two jump attempts. The best result was entered into the sheet.

#### Endurance

The Velocity Intermittent Fitness Test (VIFT–the speed achieved by the subject at the end of the test) was measured using the Intermittent Fitness Test 30–15 according to Buchheit’s assumptions (Buchheit et al., 2010). The competitors ran between lines 40 m apart, following the rhythm of sound signals. The participant’s task was to reach the designated buffer zones at a specific sound signal. Between 30 s runs at increasing speed, there were 15 s pauses of active regeneration. The initial speed of the test was set at 10 km/h. When the athlete did not run to the line twice in a row at a specific sound signal or reported fatigue, the competitor (of his own volition) ended the test. The parameter used to create the charts was MAS. This is the speed at which the athlete reaches the VO_2_ max. MAS is not directly measured in the 30–15 IFT test; VIFT is often used as a near-MAS indicator but overstated by 15%–25% due to the nature of the test. MAS is a key indicator of an athlete’s aerobic endurance and is used to plan the intensity of endurance training. For the purposes of our study, a simple estimate of the MAS value, a simplified approach, was used according to the following formula: MAS = VIFT × 0.8 (Buchheit et al., 2010).

### Statistical analysis

The Shapiro-Wilk test checked the normality of the data distribution - equivalence of variance as Levene’s test. The significance of differences between all age groups was examined with one-way ANOVA. Statistical significance was assumed at the level of *p* < 0.05. Percentile charts were created based on the division of the percentile range: P3, P10, P25, P50, P75, P90, P97. All statistical surveys were conducted using Statistica version 13.0 of TIBCO Software Inc. Results are mentioned as mean ± SD. Sample size estimated using G*Power software (version 3.1.9.2; Kiel University, Kiel, Germany) (Faul et al., 2007) returned a minimum of 35 measurement positions, for a = 0.05, effect size f = 0.8 and β = 0.95.

## Results

### Speed

**FIGURE 1:**
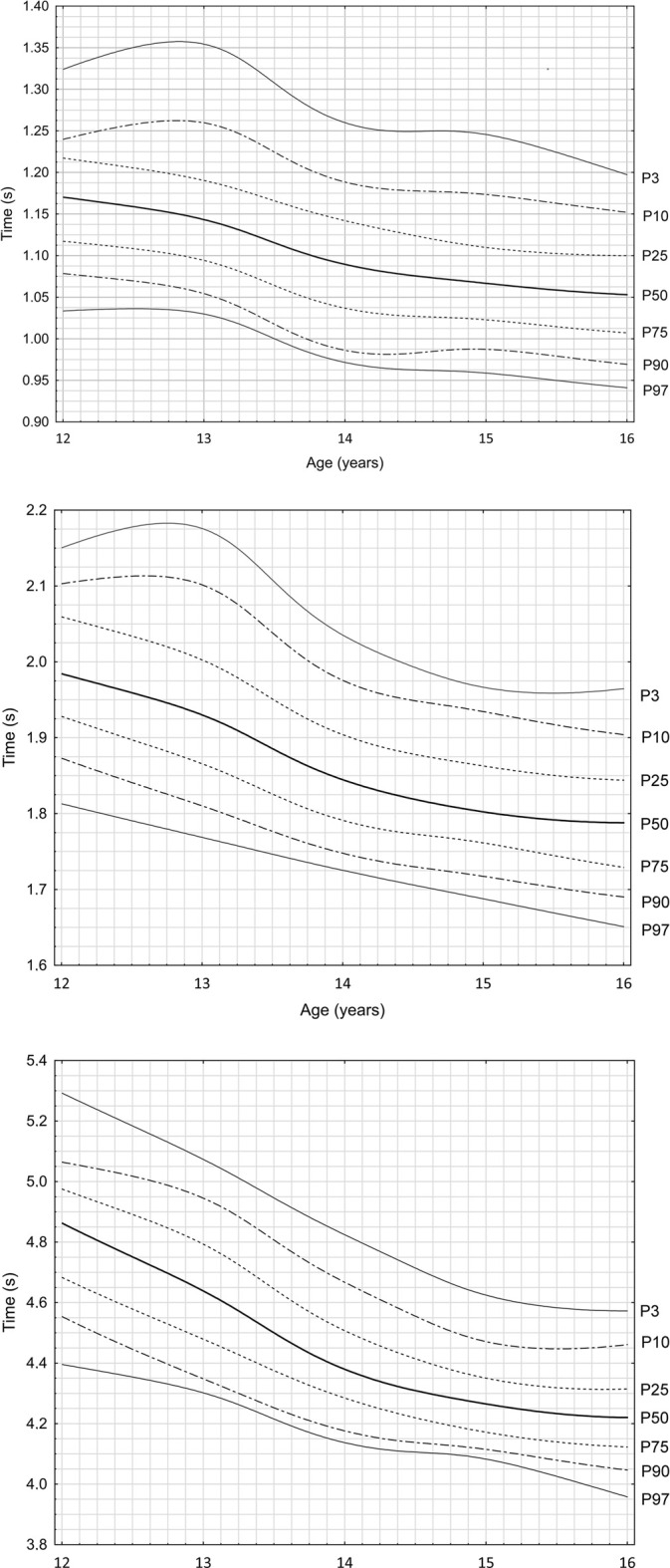
Parameter growth chart sprint time at **(A)** 5 m (F = 43.1; *p* < 0.001) for football players. *X*-axis: age of competitors (years); *Y*-axis: time over 5 m (seconds); Lines on the graph: the individual lines represent percentiles in order (P3, P10, P25, P50, P75, P90, P97). The growth charts have been developed based on data from many years of research carried out at the RKS Raków Academy. Parameter growth chart sprint time at **(B)** 10 m (F = 74.8; *p* < 0.001) for football players. *X*-axis: age of competitors (years); *Y*-axis: time over 5 m (seconds); Lines on the graph: the individual lines represent percentiles in order (P3, P10, P25, P50, P75, P90, P97). The growth charts have been developed based on data from many years of research carried out at the RKS Raków Academy. Parameter growth chart sprint time at **(C)** 30 m (F = 183.5; *p* < 0.001) for football players. *X*-axis: age of competitors (years); *Y*-axis: time over 5 m (seconds); Lines on the graph: the individual lines represent percentiles in order (P3, P10, P25, P50, P75, P90, P97). The growth charts have been developed based on data from many years of research carried out at the RKS Raków Academy.

Figures 1A–C shows the growth charts for motor skills in speed tests over running distances of 5, 10, and 30 m for young football players aged 12 to 16. The highest growth values in the scale of the entire study were: 0.126 s (P3) for 5 m; 0.215 s (P25) for 10 m; and 0.717 s (P3) for 30 m. The lowest values were: 0.087 s (P10) for 5 m; 0.162 s (P97) for 10 m; and 0.438 s (P97) for 30 m. Since the P97 value in the 5 m run is equal to 0.092 s, it can be assumed that the lowest values of positive changes were observed in the best-accelerating group of tested footballers. The intervals describing the magnitude of improvement in results at individual distances over the 5-year analysis period were as follows: for 5 m, <0.087–0.126s>; for 10 m, <0.162–0.215s>; and for 30 m, <0.438–0.719s>.

The most dynamic positive change in the value of velocity increase at all measurement distances was observed between 13 and 14 years of age, regardless of the studied percentile. The maximum magnitude of these increases was: 0.117 s (P3) for 5 m; 0.164 s (P3) for 10 m; and 0.309 s (P25) for 30 m.

In total, 84 cases were analyzed, examining changes in individual percentiles over subsequent years. In 75 cases (89%), improvement was achieved; in 4 cases (5%), stabilization of results was observed; and in 5 cases (6%), regression occurred. Detailed data are presented in Table 2.

**TABLE 2:** Values were obtained in a sprint time over 5, 10, 30 m of football players aged 12 to 16. Results are in (s) and include percentiles from P3 to P97.

Sprint time over a distance of 5 m (s)							
Percentile/Age (years)	P3	P10	P25	P50	P75	P90	P97
12	1.323	1.239	1.217	1.17	1.117	1.078	1.033
13	1.367	1.269	1.193	1.147	1.099	1.061	1.036
14	1.250	1.182	1.141	1.087	1.033	0.98	0.967
15	1.251	1.176	1.109	1.068	1.025	0.992	0.961
16	1.197	1.152	1.100	1.053	1.007	0.969	0.941

Sprint time over a distance of 10 m (s)							
Percentile/Age (years)	P3	P10	P25	P50	P75	P90	P97
12	2.149	2.102	2.059	1.984	1.928	1.873	1.813
13	2.192	2.115	2.009	1.935	1.869	1.812	1.769
14	2.028	1.967	1.900	1.842	1.788	1.746	1.725
15	1.964	1.937	1.864	1.803	1.764	1.719	1.688
16	1.965	1.904	1.844	1.788	1.729	1.690	1.651

Sprint time over a distance of 30 m (s)							
Percentile/Age (years)	P3	P10	P25	P50	P75	P90	P97
12	5.292	5.063	4.974	4.862	4.683	4.554	4.395
13	5.080	4.963	4.809	4.65	4.484	4.352	4.312
14	4.825	4.663	4.500	4.372	4.282	4.170	4.126
15	4.617	4.461	4.348	4.268	4.172	4.122	4.093
16	4.573	4.462	4.315	4.220	4.123	4.046	3.957

#### Power

**FIGURE 2:**
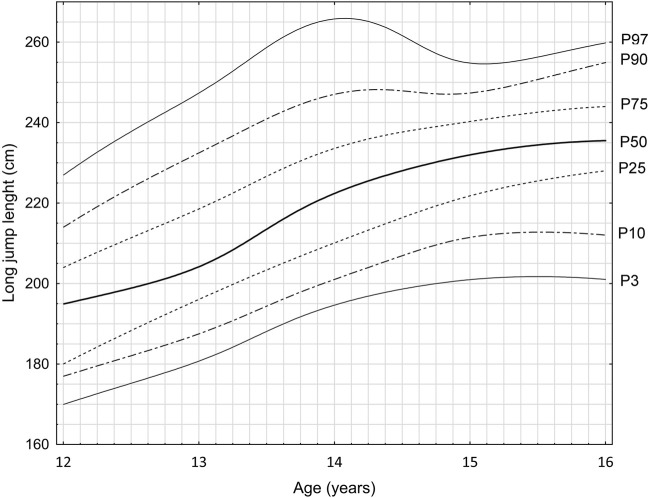
Percentile grid of the power parameter in the long jump test on the example of young football players (F = 88.6; *p* < 0.001). *X*-axis: age of competitors (years); *Y*-axis: jump length (centimeters); Lines on the graph: individual lines represent different percentiles (P3, P10, P25, P50, P75, P90, P97). The growth charts have been developed based on data from many years of research carried out at the RKS Raków Academy.

Within the lower limb power measurements, the highest level of power increase is also visible between 13 and 14 years of age. It ranges from 14 to 22 cm. The exceptions are 12–13-year-old competitors placed at the P25 and P90 percentile level, where the results are also very high and amount to 16 and 18 cm, respectively. Analyzing the differences between the percentiles in the entire process of 5-year training, the highest positive value was observed at the P25 level–an improvement of 48 cm in the long jump, while the smallest differences were visible at the P3 level – 31 cm. The range describing the magnitude of the improvement in performance over the 5-year analysis period was 31–48 cm. A total of 28 cases were analyzed, examining changes in individual percentile in between subsequent years. In 24 (86%) cases, the following were obtained: in 2 (7%) - stabilization of the result; 2 (7%) regression. Comprehensive data are presented in Figure 2, and individual averages in Table 3.

**TABLE 3:** Values obtained in the long jump test of young footballers between the ages of 12 and 16. Results are given in centimeters and include percentiles from P3 to P97.

Long jump (cm)							
Percentile/Age (years)	P3	P10	P25	P50	P75	P90	P97
12	170	177	180	195	204	214	227
13	180	187	196	203	218	232	246
14	195	201	210	223	234	248	268
15	201	212	222	232	240	246	252
16	201	212	228	235	244	255	260

#### Endurance

**FIGURE 3:**
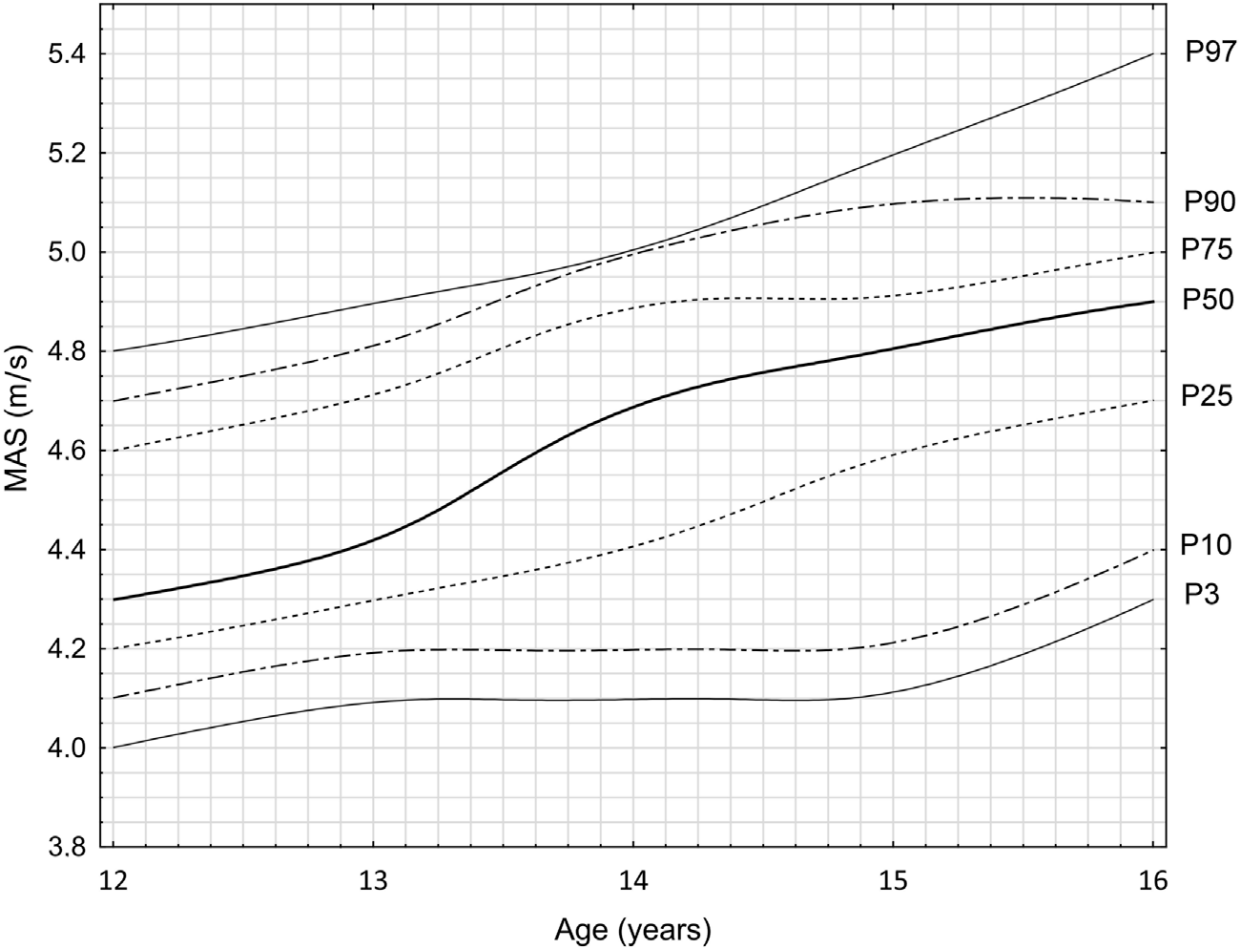
Endurance parameter growth chart in the 30–15 progressive test on the example of young football players (F = 44.3; *p* < 0.001). *X*-axis: age of competitors (years); *Y*-axis: value - Maximal Aerobic Speed (meters/seconds); Lines on the graph: individual lines represent different percentiles (P3, P10, P25, P50, P75, P90, P97). The growth charts have been developed based on data from many years of research carried out at the RKS Raków Academy.

The analysis of the endurance results indicated a different trend. Clear incremental jumps were visible between the ages of 13 and 14 at the level of–P50–0.3 m/s, P75–0.2 m/s, and P90–0.2 m/s. This constitutes 50% of the increase observed during the entire observation period. Between the ages of 14 and 15, this concerned P25 and P97–0.2 m/s–it was 40% and 33% of the entire increase, respectively. Between the ages of 15 and 16, at the level of P3 and P10–0.2 m/s – 66% of the entire increase. Considering the entire training cycle, the greatest differences were noted at the percentiles 50 and 97, with an improvement of 0.6 m/s. The lowest values are 0.3 m/s and concern percentile P3 and P10. Stagnation of the endurance parameter was also observed between the ages of 13 and 15 at the level of the two lowest percentiles, P3 and P10; the result was fixed at the level of 4.1 m/s and 4.2 m/s, respectively. The range describing the size of the improvement in the results in the perspective of 5 years of analysis was <0.3–0.6 m/s>. A total of 28 cases were analyzed, examining changes in individual percentiles between successive years. Progress was obtained in 22 (79%) cases; in 6 (21%), the result stabilized, and regression was not noted. The overall data are presented in Figure 3, and the individual averages are in Table 4.

**TABLE 4:** Values obtained in the 30–15 performance test in young footballers aged 12 to 16. Results are given in meters per second (estimated MAS value according to the formula MAS = VIFT × 0.8) and include percentiles from P3 to P97.

Maximal aerobic speed (m/s)							
Percentile/Age (years)	P3	P10	P25	P50	P75	P90	P97
12	4.0	4.1	4.2	4.3	4.6	4.7	4.8
13	4.1	4.2	4.3	4.4	4.7	4.8	4.9
14	4.1	4.2	4.4	4.7	4.9	5.0	5.0
15	4.1	4.2	4.6	4.8	4.9	5.1	5.2
16	4.3	4.4	4.7	4.9	5.0	5.1	5.4

MAS, Maximum Aerobic Speed.

## Discussion

The observed adaptive changes in motor skills in young footballers participating in the RKS Raków Academy training program based on wave periodization indicate abrupt increases in speed, power, and endurance parameters in specific age groups. This suggests that the use of wave periodization could have contributed to these increases, which allowed the development of tools for assessing the magnitude and dynamics of changes in performance parameters (Gil et al., 2007). The periodization of strength and speed training used in professional football academies is most visible at the turn of 12 and 13 years of age. Studies confirm the thesis that young footballers around 13 years of age achieve better results and more significant increase in speed and power than those who do not train in such a system (Gil et al., 2007; Malina et al., 2007).

### Speed

The most dynamic speed gains were visible in the test results between 13 and 14 years of age at 5 m, 10 m, and 30 m, regardless of percentile. This suggests that the adaptation effect in this period is particularly significant and may be crucial for developing this motor feature. The analysis of the percentage share of these increases between the age of 13 and 14 in relation to the total increases between the age of 12 and 16 for different percentiles confirms that the wave periodization model functioned most effectively in this period in the context of running speed parameters. It was effective in 89% of the analyzed cases at individual percentiles on a 5-year scale. The development of speed and power is one of the most important tasks of motor preparation coaches in football. Studies confirm the significant influence of speed and power in key moments of match competition (Rampinini et al., 2007).

The analysis showed the occurrence of a certain anomaly in the results (4 cases) at the level of the lowest percentiles, P3 and P10, in the period between the age of 12 and 13 in the 5 and 10 m tests. The regression of the results could have resulted from a large increase in body mass and height. At this stage of training, a coordination problem could have occurred, consisting in an undeveloped mechanism for effectively using the additional muscle mass in the weakest group of trainees. The solution to this problem is a better-tailored training process of an individualized nature. The works of subsequent authors highlight the need for research on biological maturity and placing players in an appropriate training system to optimize sports results (González et al., 2021; Morris et al., 2018; Philippaerts et al., 2006). The training plan for motor preparation at the RKS Raków Academy is strongly focused on using adaptation periods, which is also applicable and translated into the work of coaches according to the Long-Term Athlete Development (LTAD) program (Lloyd and Oliver, 2012).

### Power

In lower limb power measurements, the highest increases were observed between 13 and 14 years of age, ranging from 8 to 14 cm and 14–22 cm. In the remaining years, the increases are smaller, or the results stabilize at one level. Similar observations were made by Szymanek-Pilarczyk et al. (2023), where the minimum and maximum results in the long jump tests stabilized at a level from about 190 cm to 287 cm in 15 and 16-year-olds. Although the increases in muscle mass were not directly analyzed, it can be assumed that the increased muscle mass during this period contributed to the increase in power. The increase in power is crucial for activities such as acceleration or changes in direction (De Salles et al., 2012). For coaches, this means that training explosive lower limb strength should be a priority at this age. From a scientific perspective, the greatest increases in power at the P25 percentile level suggest that average-level players have great potential for improvement with appropriate training. The research by Skratek et al. (2024) suggests that long-term, traditional strength training is effective and safe for young soccer players between the ages of 12 and 15 and should be included in long-term athletic development programs. This emphasizes the importance of individualizing the training process (Skratek et al., 2024; Szymanek-Pilarczyk et al., 2023).

### Endurance

The MAS coefficient, which was one of the parameters studied in this study, is highly correlated with running efficiency, which is very important in football. This parameter allows for determining the speeds at which the player works using maximum oxygen consumption, thus determining the training load (Buchheit et al., 2021). A similar trend to that observed among the Częstochowa Academy footballers can be observed in the studies conducted by Deprez et al. (2015a). Players who initially achieved very high results in the following years also achieved very high results (clear incremental jumps). This is confirmed by the observation of the behavior of this parameter at the level of the P97 percentile, where the increase in the MAS parameter value was 0.6 m/s between the ages of 12 and 16, as well as the difference in the values of the P25 and P97 percentile in individual years amounting to 0.6 m/s, and at the age of 16 even 0.7 m/s between P25 and P97.

Most of the analyzed subjects noted a positive adaptation to the proposed work system called wave periodization. The studies observed that there is a group of players from the P3 and P10 levels whose unplanned stability of results was observed - this concerns players aged 13 to 15. Their results remained at the level of 4.1 m/s and 4.2 m/s, respectively. It can be argued that this feature is strongly determined by genetics, and this type of training does not positively affect the development of aerobic capacity for this group of players. However, no scientific studies show that this excludes them from being effective and useful football players. It also opens up space for the coach to manage such a group of players differently (Deprez et al., 2015b). Moreover, many variables can disturb or distort the linear development of MAS speed. Starting from a growth spurt and weight fluctuations to changes in position during the training process, which is associated with other values achieved on the morphocycle scale (Abbott and Collins, 2002). The research conducted by Nobari et al. confirms how the position on the pitch determines the development of such parameters as MAS and VO2max (Nobari et al., 2021).

It is very important to skillfully use the results in the percentile grids dedicated to the appropriate sports level (Murtagh et al., 2018). In youth football, the requirements for players in many areas are constantly changing. The position of the first teams in the table is related to their financing, which directly affects the development possibilities of the academy through the prism of multi-year training. Regulations regarding the stability of such projects would have a positive impact on their harmonious development in the long term. Therefore, such a tool can help build stable foundations and programs for youth football. The task of the authors of the work is to ensure a calm, smooth development of players, without the so-called “disappearance” in the system, and to prepare patterns for introducing young, outstanding players to senior football. This research shows that players achieving low results can also catch up with potentially better ones if an appropriately selected, individualized training program is applied to them. The resulting percentile grids can facilitate the prediction of player development in many areas (Reilly et al., 2000). Using the information in the percentile charts developed during many years of research and considering the Peak Height Velocity (PHV) period may additionally help optimize the training process, achieve training effects, and gradually reduce the risk of injury (Myer et al., 2011).

### Practical application

Percentile charts, developed based on research data, constitute a useful tool for coaches and other specialists in the field of physical education sciences, such as physiotherapists and doctors, to monitor and assess the development of motor abilities in young football players. Their regular use allows for the individualization of the training process and the adjustment of loads and training methods to the needs of each athlete, which can contribute to the optimization of training outcomes and the reduction of injury risk (Myer et al., 2011).

Establishing normative value results enables precise identification of potential gaps in the sports specialization of athletes. Percentile charts can serve as a reference point for a given football level and be helpful in planning the return-to-sport stage after injuries. Such a tool facilitates planning work with youth, where the introduction of systematic monitoring and individualization is necessary, positively impacting the improvement of the training level.

### Limitations

The main limitation is the lack of direct correlation analysis of muscle mass gains and other factors, such as changes in position on the pitch or individual differences in biological maturation—precise measurement of PHV. The age range does not include 17- and 18-year-olds. In addition, the study concerned only one football academy, which may limit the possibility of generalizing the results to a wider population of young footballers.

### Future research directions

Future research directions include the creation of similar growth charts for males and females aged 8 to 18. This will provide a more comprehensive understanding of the development of motor skills in young athletes regardless of gender and age. This type of research will allow for even more precise adjustment of training programs and monitoring of the progress of players in different age and gender groups, which in the long term will contribute to raising the level of sports performance of all young football players. An intermediate goal is to create an open spreadsheet, updated every year and made available to coaches to compare the sports results of children and young people. In addition, the development of a publicly available tool will allow academies worldwide to use similar tests to compare the results of their players. This will enable global standardization of results, which will contribute to a better understanding and optimization of the training process of young football players around the world.

Players’ physical attributes, such as height, weight, and speed, can affect their suitability for different positions on the pitch. Taller and stronger players may be better suited for defense, while faster and more agile players may be better suited for attacking. Further research into these relationships could provide valuable insights into talent identification, tactical planning, and improved positional matching.

## Conclusion

1. The greatest increases in lower limb power and speed occur between the ages of 13 and 14, which indicates the need for intensified training of these features at this age.
2. Differences in motor skill gains between athletes at different percentiles emphasize the need for individualized training, especially for athletes at the highest and lowest levels.
3. The use of the wave periodization model can effectively support the development of motor skills when it is adapted to the individual pace of maturation of athletes.
4. Percentile charts are a valuable tool for coaches to monitor progress and plan individual training programs, which can lead to better sports results.

## Data Availability

The raw data supporting the conclusions of this article will be made available by the authors, without undue reservation.
