# Supplementary material for: Normative and limit values of speed, endurance and power tests results of young football players
Source: Front Physiol. 2025 Jan 8;15:1502694. doi: 10.3389/fphys.2024.1502694 (PMC11751035; doi:10.3389/fphys.2024.1502694)
Supplement: Supplementary file 1 [file Table1.docx]

| **Age**  **(years)** | **Number of players** | **Body height (cm) ± SD** | **Body weight (kg) ± SD** | **Body fat (%) ± SD** | **Muscle mass (kg) ± SD** |
| --- | --- | --- | --- | --- | --- |
| **16** | 98 | 178 ± 3.89 | 69.02 ± 5.17 | 15.15 ± 1.97 | 54.75 ± 4.18 |
| **15** | 102 | 178 ± 4.71 | 67.20 ± 5.42 | 15.22 ± 1.92 | 54.05 ± 4.42 |
| **14** | 101 | 172 ± 7.00 | 59.30 ± 6.78 | 14.99 ± 2.39 | 47.74 ± 4.73 |
| **13** | 97 | 165 ± 9.42 | 51.27 ± 8.91 | 15.15 ± 2.69 | 40.89 ± 6.82 |
| **12** | 97 | 154 ± 4.59 | 42.83 ± 7.11 | 19.24 ± 6.62 | 32.12 ± 4.71 |

Table 1. The characteristics of the study group are divided into age categories.
