# Supplementary material for: Normative and limit values of speed, endurance and power tests results of young football players
Source: Front Physiol. 2025 Jan 8;15:1502694. doi: 10.3389/fphys.2024.1502694 (PMC11751035; doi:10.3389/fphys.2024.1502694)
Supplement: Supplementary file 2 [file Table2.docx]

| **Sprint time over a distance of 5m (s)** | | | | | | | | |
| --- | --- | --- | --- | --- | --- | --- | --- | --- |
| P**ercentile / Age (years)** | **P3** | **P10** | **P25** | **P50** | **P75** | **P90** | **P97** |  |
| **12** | 1.323 | 1.239 | 1.217 | 1.17 | 1.117 | 1.078 | 1.033 |  |
| **13** | 1.367 | 1.269 | 1.193 | 1.147 | 1.099 | 1.061 | 1.036 |  |
| **14** | 1.250 | 1.182 | 1.141 | 1.087 | 1.033 | 0.98 | 0.967 |  |
| **15** | 1.251 | 1.176 | 1.109 | 1.068 | 1.025 | 0.992 | 0.961 |  |
| **16** | 1.197 | 1.152 | 1.100 | 1.053 | 1.007 | 0.969 | 0.941 |  |
| **Sprint time over a distance of 10m (s)** | | | | | | | | |
| P**ercentile / Age (years)** | **P3** | **P10** | **P25** | **P50** | **P75** | **P90** | **P97** |  |
| **12** | 2.149 | 2.102 | 2.059 | 1.984 | 1.928 | 1.873 | 1.813 |  |
| **13** | 2.192 | 2.115 | 2.009 | 1.935 | 1.869 | 1.812 | 1.769 |  |
| **14** | 2.028 | 1.967 | 1.900 | 1.842 | 1.788 | 1.746 | 1.725 |  |
| **15** | 1.964 | 1.937 | 1.864 | 1.803 | 1.764 | 1.719 | 1.688 |  |
| **16** | 1.965 | 1.904 | 1.844 | 1.788 | 1.729 | 1.690 | 1.651 |  |
| **Sprint time over a distance of 30m (s)** | | | | | | | | |
| P**ercentile / Age (years)** | **P3** | **P10** | **P25** | **P50** | **P75** | **P90** | **P97** |  |
| **12** | 5.292 | 5.063 | 4.974 | 4.862 | 4.683 | 4.554 | 4.395 |  |
| **13** | 5.080 | 4.963 | 4.809 | 4.65 | 4.484 | 4.352 | 4.312 |  |
| **14** | 4.825 | 4.663 | 4.500 | 4.372 | 4.282 | 4.170 | 4.126 |  |
| **15** | 4.617 | 4.461 | 4.348 | 4.268 | 4.172 | 4.122 | 4.093 |  |
| **16** | 4.573 | 4.462 | 4.315 | 4.22 | 4.123 | 4.046 | 3.957 |  |

Table 2. Values were obtained in a sprint time over 5, 10, 30m of football players aged 12 to 16. Results are in (s) and include percentiles from P3 to P97.
