# Supplementary material for: Normative and limit values of speed, endurance and power tests results of young football players
Source: Front Physiol. 2025 Jan 8;15:1502694. doi: 10.3389/fphys.2024.1502694 (PMC11751035; doi:10.3389/fphys.2024.1502694)
Supplement: Supplementary file 3 [file Table3.docx]

| **Long jump (cm)** | | | | | | | |
| --- | --- | --- | --- | --- | --- | --- | --- |
| P**ercentile / Age (years)** | **P3** | **P10** | **P25** | **P50** | **P75** | **P90** | **P97** |
| **12** | 170 | 177 | 180 | 195 | 204 | 214 | 227 |
| **13** | 180 | 187 | 196 | 203 | 218 | 232 | 246 |
| **14** | 195 | 201 | 210 | 223 | 234 | 248 | 268 |
| **15** | 201 | 212 | 222 | 232 | 240 | 246 | 252 |
| **16** | 201 | 212 | 228 | 235 | 244 | 255 | 260 |

Table 3. Values obtained in the long jump test of young footballers between the ages of 12 and 16. Results are given in centimeters and include percentiles from P3 to P97.
