# Supplementary material for: Normative and limit values of speed, endurance and power tests results of young football players
Source: Front Physiol. 2025 Jan 8;15:1502694. doi: 10.3389/fphys.2024.1502694 (PMC11751035; doi:10.3389/fphys.2024.1502694)
Supplement: Supplementary file 4 [file Table4.docx]

| **Maximal Aerobic Speed (m/s)** | | | | | | | | |
| --- | --- | --- | --- | --- | --- | --- | --- | --- |
| P**ercentile / Age (years)** | **P3** | **P10** | **P25** | **P50** | **P75** | **P90** | **P97** |  |
| **12** | 4.0 | 4.1 | 4.2 | 4.3 | 4.6 | 4.7 | 4.8 |  |
| **13** | 4.1 | 4.2 | 4.3 | 4.4 | 4.7 | 4.8 | 4.9 |  |
| **14** | 4.1 | 4.2 | 4.4 | 4.7 | 4.9 | 5.0 | 5.0 |  |
| **15** | 4.1 | 4.2 | 4.6 | 4.8 | 4.9 | 5.1 | 5.2 |  |
| **16** | 4.3 | 4.4 | 4.7 | 4.9 | 5.0 | 5.1 | 5.4 |  |

Table 4. Values obtained in the 30-15 performance test in young footballers aged 12 to 16. Results are given in meters per second (estimated MAS value according to the formula MAS = VIFT × 0.8) and include percentiles from P3 to P97.
